# James German and the Quest to Understand Human RECQ Helicase Deficiencies

**DOI:** 10.3390/cells13131077

**Published:** 2024-06-21

**Authors:** Raymond J. Monnat

**Affiliations:** Departments of Laboratory Medicine/Pathology and Genome Sciences, University of Washington, Seattle, WA 98195, USA; monnat@uw.edu; Tel.: +1-206-616-7392

**Keywords:** Werner syndrome, Bloom syndrome, RECQ helicase deficiency syndrome, progeroid syndrome, cancer predisposition syndrome

## Abstract

James German’s work to establish the natural history and cancer risk associated with Bloom syndrome (BS) has had a strong influence on the generation of scientists and clinicians working to understand other RECQ deficiencies and heritable cancer predisposition syndromes. I summarize work by us and others below, inspired by James German’s precedents with BS, to understand and compare BS with the other heritable RECQ deficiency syndromes with a focus on Werner syndrome (WS). What we know, unanswered questions and new opportunities are discussed, as are potential ways to treat or modify WS-associated disease mechanisms and pathways.

## 1. Introduction

**Figure 1:**
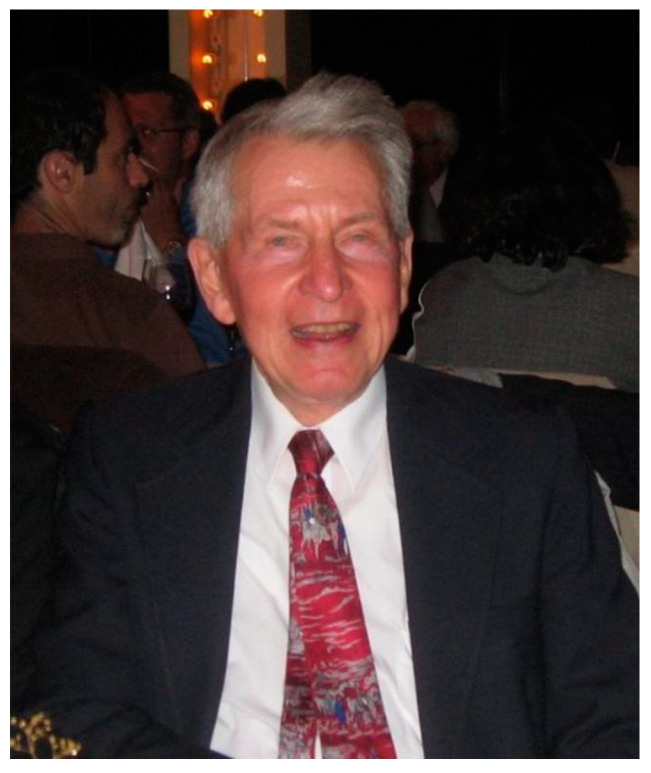
James German (1926–2018); 2009 photo by Dr. Raymond J. Monnat, Jr., University of Washington.

An excellent sense of James German’s intellectual history and trajectory is provided by the candid, discursive reminiscence he wrote to honor his mentor and friend Henry Kunkel [1] (Figure 1). James’ thinking and early work on Bloom syndrome (BS) are captured in more detail in ‘*Chromosome Mutation and Neoplasia*’ [2], a 1983 monograph James organized and edited. This monograph included chapters by James on cytogenetics, and on cancer associations of the chromosome breakage syndromes including a detailed look at BS. James also wrote a perceptive monograph introduction focused on themes that continue to guide work and thinking today: that karyotypic—that is, chromosome-level—data have the ability to provide continued new insight into the origins and progression of cancer; and that neoplasia is the outcome of the universal and fundamental biological process of clonal evolution. These related, larger themes were further developed in ‘*Chromosome Mutation*’, with chapters contributed by John Cairns, who focused on familial defects as a way to investigate cancer origins, and by Peter Nowell, who focused on clonal evolution in neoplastic progression.

## 2. Getting Started Thinking about Human Disease

Diseases, especially heritable syndromic diseases, are nature’s way of drawing attention to important, though often hidden, aspects of human biology. These ‘Experiments of Nature’ represent powerful ways to gain insight into disease origins and mechanisms, and are equally fascinating and remarkable in their ability to capture imaginations and entire careers: witness James German’s decades-long pursuit of Bloom syndrome! Bloom syndrome (BS) is a heritable, recessive genetic instability and cancer predisposition syndrome caused by bi-allelic loss of function mutations in the *BLM* gene that encodes one of the five human RECQ helicase proteins. The BS phenotype was first clearly described in 1954 by David Bloom as a ‘…congenital telangiectatic erythema resembling lupus erythematosus in dwarfs’ that was likely a syndrome entity, and was striking: proportional dwarfing, often a butterfly distribution facial skin rash and immune deficiency with a remarkably high risk of developing a broad spectrum of different histologic types of cancer [3]. Other human RECQ helicase deficiency syndromes have been recognized, though again were not clearly linked to the human *RECQ* helicase gene family prior to gene positional cloning and mutation analyses. These include Werner syndrome (WS), caused by loss-of-function mutations in the *WRN* gene and a primary focus for this perspective; a subset of Rothmund-Thomson syndrome caused by loss-of-function mutations in the *RECQL4* gene [4,5]; and the recently identified RECON progeroid and genomic instability syndrome caused by biallelic loss-of-function mutations in the *RECQL* gene [6].

**Figure 2:**
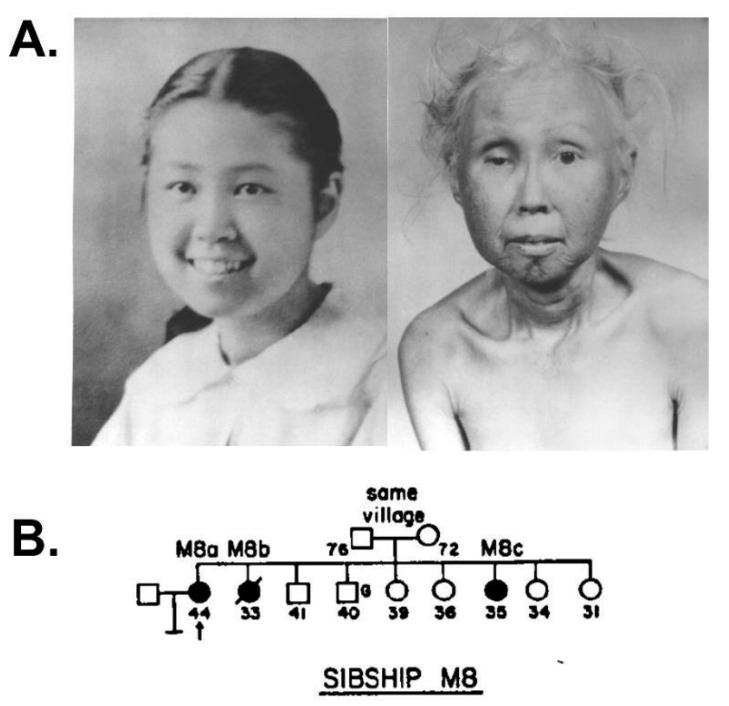
Werner syndrome clinical phenotype and progression. (**A**) Photos of WS patient M8a at ages 15 and 48 illustrate WS progression of physical signs over three decades. These provide a graphic example of why WS is often referred to as an adult ‘premature aging’ syndrome. (**B**) WS patient M8a shown in panel A was aged 44 (pedigree arrow) when first seen in 1960 as one of three affected sisters in a Japanese–American family examined by Arno Motulsky in the Medical Genetics Clinic at the University of Washington in Seattle. Source notes: The photos in A were scanned and digitally restored from original patient photos provided to the author by George Martin and Nancy Hanson of the Werner Syndrome International Registry, who gave permission for their reuse. (**B**) is from Figure 13 of Epstein et al., 1966 [8].

**Figure 3:**
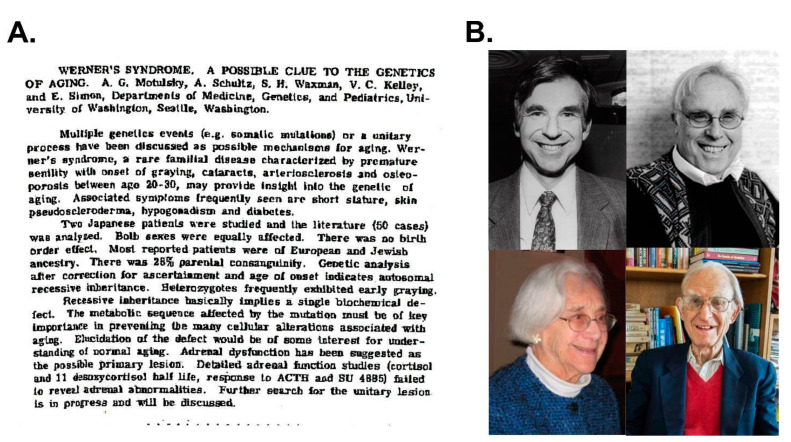
Initial reporting of the ‘rediscovery’ of WS. (**A**) Abstract presented by Arno Motulsky at the 1961 American Society of Medical Genetics meeting in Atlantic City. Notably, James German presented a case report the previous evening, on a Down syndrome patient with leukemia and a chromosomal translocation. (**B**) Co-authors of the seminal, comprehensive 1966 review on WS [8]. Arno Motulsky, in a 2002 personal communication to the author, noted that Amelia Schultz, the least well-known of the co-authors, ‘…was a PhD anthropologist who had also trained as a social worker. When we started the Genetics Clinic in 1959 (when the University Hospital started), she was a great help with patients and helping with the Werner Syndrome literature’ (see [9] for additional detail). Source notes: (**A**) ASHG abstract copy provided to the author by Arno Motulsky in 2002. (**B**) Photos of 1966 WS review co-authors, who all led remarkable, event-filled lives: Charles Epstein (upper left, photo from his NY Times obituary); George Martin (upper right, photo courtesy Department of Pathology, University of WA); Amelia Schultz (lower left, from Miller [9]); and Arno Motulsky (lower right, photo from his NY Times obituary).

Clinical photos and patient case series emphasize that WS has a striking, though substantially different clinical presentation and phenotype than BS (Figure 2) [3,7]. The features of WS reminiscent of accelerated aging have led to its being characterized as the ‘prototypical heritable adult “premature aging” syndrome’. This aspect of WS (Figure 3) stimulated considerable interest in the idea that a single gene defect might provide broad new insight into the biology of aging, and reveal some of the deeper links between aging, age-associated disease pathogenesis and cancer risk. These ideas came into sharper focus when I moved to the University of Washington in Seattle to complete my medical training and, as I relate below, provided many new opportunities to pursue both clinical and more basic biological questions about WS.

From the initial pedigree in Figure 2, local colleagues carried out a comprehensive descriptive analysis of WS that integrated local patient data, clinical case reports and analyses of pathology material from WS patient autopsies. Arno Motulsky provided as part of this manuscript a formal genetic pedigree analysis that established the autosomal recessive inheritance of WS [8].

The formal genetics and pedigree analysis of WS indicated that the pleiotropic clinical phenotype of WS was being driven by the loss of function of a single gene. The importance of this inheritance pattern was not lost on Arno Motulsky, who wrote: ‘Recessive inheritance basically implies a single biochemical defect. The metabolic sequence affected by the mutation must be of key importance in preventing the many cellular alterations associated with aging. […] When Charles Epstein with a superior biochemistry background came as a fellow in 1964, I hoped he might find the gene. It took another 30 years or so!’ (A. Motulsky, 2002, personal communication). These tantalizing clinical features of WS made it ripe for molecular attack to identify the associated disease gene, syndrome-associated mutations and how loss of function might catalyze such a wide range of phenotypic consequences (Figure 2).

## 3. Werner Syndrome—First Steps, and a Disease Gene Cloning ‘Prequel’

Prior to 1996, most research on WS focused on better defining the clinical phenotype and natural history, and on cellular, molecular and biochemical phenotypes. These paralleled at several points the important early precedent established for BS, that revealed a characteristic cytogenetic instability phenotype in BS consisting of elevated sister chromatid exchanges, quadriradials and chromatid breaks, gaps and telomere associations; all of these cytogenetic features suggested a defect in recombination in somatic cells [10]. WS fibroblasts displayed a markedly limited replicative lifespan in vitro [11] and a chromosomal instability phenotype described as ‘variegated translocation mosaicism’ [12]. These and many other ‘pre-genomic’ analyses and clues to the biology of WS were summarized in proceedings from a December 1982 ‘U.S.–Japan Cooperative Seminar on Werner Syndrome and Human Aging’ held in Kobe, Japan [13]. This meeting reflected the growing collaborative connections between Japanese and American colleagues that enabled cloning of the *WRN* locus, and additional, still-ongoing collaborative efforts focused on WS disease associations, pathogenesis and patient care.

## 4. 1996—The Werner Syndrome Positional Cloning ‘Annus Mirabilis’

It is hard to appreciate how competitive—and how difficult—disease gene cloning was without the genomic tools and resources we now take for granted. The hunt for the *WRN* Werner syndrome gene was all the more pressing in light of the seminal publication in 1995 of the cloning of the BS-associated gene *BLM* that was immediately identified as a member of the human *RECQ* gene family [14]. Feverish work to clone the *WRN* locus proceeded here and elsewhere with a degree of secrecy, paranoia and uncertainty, all amid constant reminders that this was more or less a ‘zero sum’ game: ‘winners’ took most, if not all, of the prize, as once a putative ‘disease gene’ was in hand, there were many immediate and well-established ways to advance understanding.

**Figure 4:**
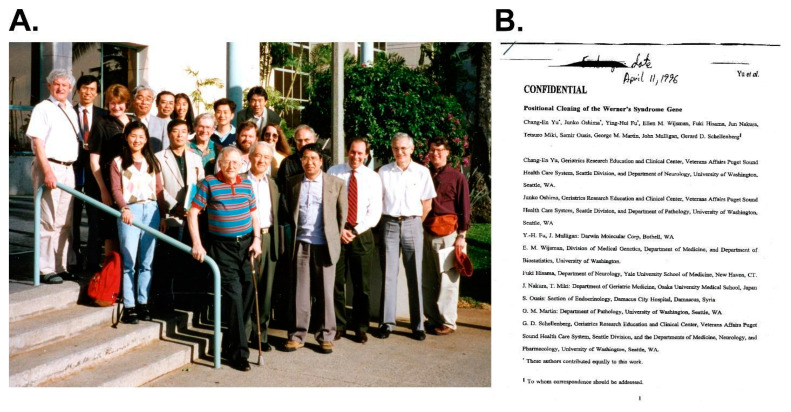
U.S.–Japan Cooperative Cancer Program Meeting 1996—First reporting and (somewhat open) discussion of *WRN* locus positional cloning. (**A**) Participants in a U.S.–Japan Cooperative Cancer Research Program meeting held in early 1996 in Honolulu to discuss WS, organized by Bob Miller (front center in a striped polo shirt, hand on rail) and Haruo Sugano (to Bob Miller’s immediate left) of the Japanese National Cancer Institute. Makoto Goto is to Haruo Sugano’s left in the front row, with Yuichi Ishikawa in the back row 2nd from the left; the author is far right with hat in hand. (**B**). Embargoed *Science* manuscript reporting *WRN* locus cloning and identification of WS disease-causative pathogenic variants, with partially crossed out embargo end date of 11 April 1996, just after the U.S.–Japan Cooperative meeting ended. Source notes: meeting photo and embargoed manuscript from the author’s archive.

George Martin laid much of the groundwork for the *WRN* locus positional cloning effort in Seattle by developing the Werner Syndrome International Registry in 1992 [15]; by assembling a growing collection of clinical material developed in collaboration with Japanese and other colleagues; and by facilitating the development and use of homozygosity mapping to convert an initial linkage assignment of *WRN* to chromosome 8 into molecular clones that enabled gene finding and the identification of putative loss of function mutations, as predicted by the formal WS recessive inheritance pattern [16,17]. Much of the flavor of this period has been captured in a short history written by George Martin in 2001 [18], in which he covers the fateful, somewhat awkward meeting of competing positional cloning teams in early 1996 as part of another U.S.–Japan Cooperative meeting held in Honolulu (Figure 4). Both of the competing positional cloning groups strongly hinted that they had succeeded, although it was clear each had identified different genes located on the short arm of chromosome 8! We were confident we had succeeded in both our gene identification and the delineation of WS-causative pathogenic *WRN* variants but were under a strict publication embargo from *Science* that prevented us from openly discussing our results (Figure 4).

Bob Miller of the NCI played a key role as a senior leader in the NCI’s Genetic Epidemiology branch in recognizing and developing the many clinical and research opportunities presented by WS and other heritable cancer predisposition syndromes. He pursued these in part by organizing the U.S.–Japan Cooperative meetings that first brought together Makoto Goto, a Japanese colleague with a great deal of experience with WS, and other investigators focused on cancer predisposition syndromes. The U.S.–Japan Cooperative Cancer Research Program was an important crossroads for sharing early work on WS and many other aspects of cancer biology and genetics, and a tribute to the foresight and friendship of Bob Miller and Haruo Sugano of the Japanese Cancer Institute [19].

Bob Miller also strongly encouraged efforts to better define the cancer predisposition in WS and the related RECQ helicase deficiency syndromes. The goal here was guided in part by the pioneering work of James German to better define BS natural history and the associated cancer histologic type-specific risk [10]. The group that embarked on this project included my lab in Seattle, Yuicihi Ishikawa, a Japanese pathology-trained colleague who had already published initial descriptions of cancer in WS [20,21] and Tom Vaughan of Fred Hutchinson/UW Cancer Center Epidemiology Program. Despite the clarity of the goal, it took several years to complete this project in a way that gave rigorous and useful results. This effort was led by Julia Lauper, a brilliant and determined University of Washington Epidemiology Master’s degree student, with help from a gifted medical student fluent in Japanese and Hideaki Tsukuma of the Osaka Cancer Registry, who identified appropriate Japanese population control data to allow quantitative cancer-type-specific risk estimations. This analysis identified five cancer types in WS patients that by two standard epidemiological risk criteria displayed elevated type-specific risks in WS patients: elevated risks ranged from 54-fold for acral and mucosal melanomas to a nearly 9-fold elevated risk for thyroid follicular epithelial neoplasms in Japan-resident WS patients versus Osaka prefecture population controls [22].

## 5. WRN and Other RECQ Macromolecular Machines

Positional cloning of the five human *RECQ* helicase genes beginning in 1994 catalyzed a wide range of work to define key biochemical and cell biological aspects of all five human RECQ helicase proteins. These efforts brought in many talented investigators with biochemical and cell biological expertise, who rapidly grew the nascent field of RECQ helicase science, including the late Judy Campisi in Berkeley; Nathan Ellis and Joanna Groden in New York; my local UW colleagues Larry Loeb, Junko Oshima and Nancy Maizels; Curtis Harris at the NCI; and Vilhelm Bohr and Bob Brosh at the NIA, among many other investigators.

Specific contributions made by members of my lab and by Seattle colleagues included biochemical characterization of the activity, polarity and substrate preferences of WRN [23,24,25]; quantifying WRN protein expression in patient-derived cell lines, using purified recombinant protein as a molecular copy number standard [26]; measuring genetic instability in vivo in genotyped members of WS kindreds using the red blood cell-based glycophorin-A assay with the help of Livermore colleagues [27]; and demonstrating a role for WRN in limiting MYC-induced cellular senescence [28]. Another conceptually important result was the identification of a mitotic recombination resolution defect in WS by using integrated mitotic recombination reporters [29,30], and a demonstration using missense substitutions in the WRN helicase and exonuclease domains that both biochemical activities were required for high cell viability after DNA damage and successful homology-dependent recombination resolution [30,31]. This last observation was satisfyingly consistent with the observed recessive inheritance of WS and the observation that no clinically ascertained WS patient has been reported with selective loss of only WRN exonuclease or helicase activity. The identification of WS as a human disease linked to a mitotic recombination resolution defect was quickly followed by related work that identified a distinct recombination resolution defect in BS, and provided a better mechanistic understanding of how these related but distinct defects led to the distinct cytogenetic signatures of BS and WS [32]. These discoveries were also important milestones in eventual identification of the mitotic recombination resolution machinery of human somatic cells [33,34].

Replication defects were also identified in early analyses of WS and BS cells [35,36,37,38]. These made perfect sense in retrospect, as preferred DNA substrates for RECQ helicases are generated by many key DNA metabolic processes [39]. High resolution quantitative assays have provided more recent mechanistic insight into these replication defects and associated repair defects in WS and BS cells. My longtime colleague and collaborator Julia Sidorova brought considerable new light to these questions in both WS and BS by developing a simple microfluidic platform (‘maRTA’, for microfluidic-assisted Replication Track Analysis) to perform faster, simpler and much more reproducible DNA ‘combing’ assays to generate quantitative, high-resolution single molecule replication and repair data on living cells [40]. This assay in different formats can report direct measures of replication such as DNA chain growth rates, fork asymmetry and the probabilities of fork stalling and restart [40], and was used to reveal distinct roles for WRN, BLM, RECQL4 and RECQ1 in replication fork assembly, progression, arrest and recovery prior to and after HU-mediated arrest or other types of DNA damage [40,41,42,43].

A chance meeting with NCI colleague Curtis Harris opened another productive, continuing line of research. Curt had an early interest in the RECQ helicases, and contributed important work to define interactions of WRN with other key cellular proteins such as TP53 [44,45,46]. Over lunch at the 2011 Aspen Cancer Conference, we found our labs were independently pursuing gene expression profiling analyses in BS and WS patient-derived fibroblasts, and in control fibroblasts acutely depleted of WRN or BLM protein. We quickly saw the benefit of pooling our complementary efforts, and worked to complete a pair of manuscripts based on comprehensive expression profiling of genes and miRNAs in WS and BS-deficient cells [47,48].

This work clearly distinguished WS and BS as related, though transcriptionally distinct, disease states by virtue of different overlapping sets of genes and miRNAs whose expression was WRN- or BLM-responsive. These experiments also emphasized long-suspected significant differences in the cell states that accompany heritable loss of WRN or BLM, or acute depletion of either protein from primary fibroblasts, a clinically affected cell lineage in both WS and BS. These gene expression data remain an important resource for both WS and BS science and have important implications for both disease modeling and disease pathogenesis, as discussed below. This collaborative project also provided a great counterexample to an oft-quoted adage by showing it is indeed possible—not to mention faster and a lot more fun—to work together rather than attempting this type of journey alone! This project and other work throughout this period reflected a strong experimental prejudice I picked up from listening to my friend and colleague Jim Haber. Jim showed how to build a productive and revealing research program by focusing on ‘in vivo biochemistry’, a term he invented to describe the use of mechanistically informative assays to determine what a given gene, protein or substrate can—and cannot—do in living cells. These types of data allow for real biological insight [49]. When we could not use this approach, we turned to direct measures to test hypotheses or ideas that often utilized WS patient autopsy tissue, histopathology blocks or slides with relevant tissue controls (see, e.g., [50]).

## 6. Broader Roles for WRN in Sporadic Cancer

A provocative paper published in 1996 led us to pursue another productive, clinically important line of WS research: Esteller and colleagues claimed that *WRN* gene silencing was common in human epithelial malignancies, and that WRN loss was a major determinant of treated outcomes in advanced stage colorectal cancers [51]. If true, we realized that this would be a very big story in cancer epigenetics, and thus set out with Dutch colleagues to replicate the findings. Alas, we were not able to confirm the initial report of widespread *WRN* methylation and inactivation, or an association with better treated outcomes. However, we did identify a more interesting and revealing story that RECQ helicase over-expression contributes to tumor survival, which is a focus for translational work on colorectal and other cancers [52,53,54,55]. Subsequent work has revealed a broader role for WRN in many cancers and uncovered therapeutically useful synthetic interactions. For example, work we participated in as part of a Cancer Genome Pan-Cancer Atlas DDR (DNA Damage and Response) Working Group identified *WRN* as one of the Top 50 altered DDR genes among the >10,000 cancers included in the Pan Cancer Atlas cohort [56].

**Figure 5:**
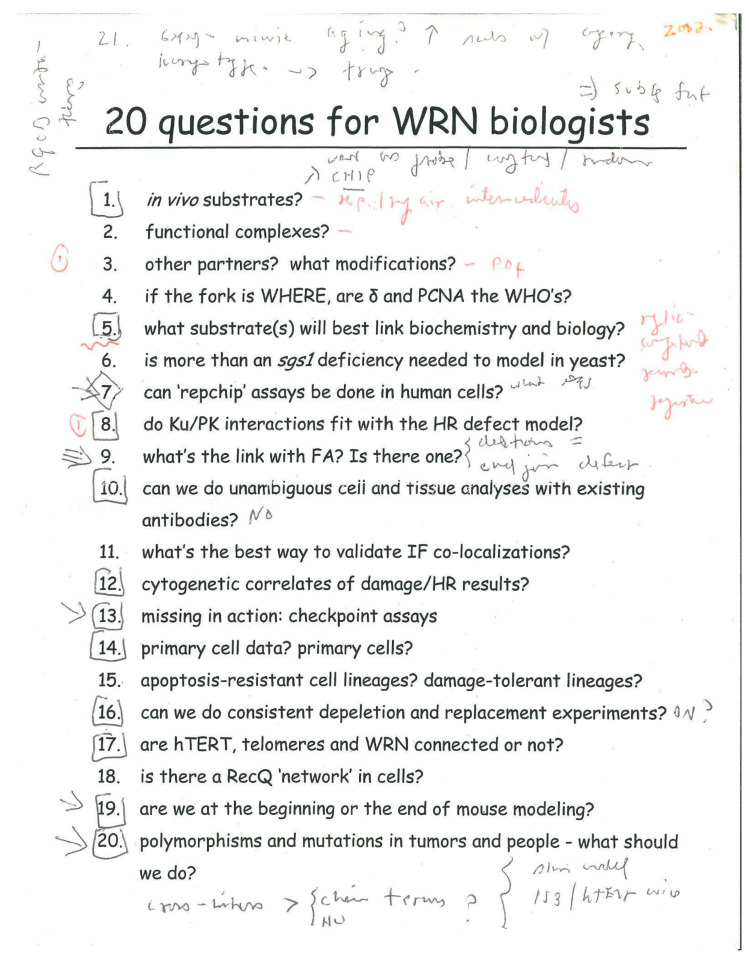
‘20 questions for WRN biologists’: This list, covered with (now largely cryptic!) handwritten notes and comments, documents the animated and extended discussion with colleagues and visitors as part of a WS-focused research retreat held in Seattle in 2002. Source: author’s archive.

Shortly after these Pan-Cancer Atlas results were published, several groups identified a synthetic lethal interaction between WRN loss and mismatch repair deficiency in human epithelial cancers [57,58,59] [reviewed in Chan 2022 monograph]. This observation led to a flurry of work to identify potent, specific WRN inhibitors in addition to the original small molecule inhibitors identified by Bob Brosh and colleagues [60,61]. Subsequent work has provided some mechanistic insight by identifying a role of WRN in resolving large palindromic AT-rich DNA sequences [62,63,64]. Our earlier work on G4-forming DNA sequences as WRN- and BLM-dependent transcription regulatory DNA elements [37,38] focused on GC-rich, as opposed to AT-rich, sequences, so we have some way to go to fully integrate these two stories into a single mechanistic framework. These few highlights emphasize important experimental milestones and important new conceptual and practical findings. It is nonetheless humbling to look back at the list of ’20 questions for Werner biologists’ compiled in 2002, and to realize how few satisfying answers we yet have to even seemingly simple questions! (Figure 5).

## 7. New Conceptual and Experimental Growing Points

How do we or should we advance the story above to better understand WS, other RECQ deficiency syndromes and their relationship to cancer and aging? Here are potential directions and ideas that I hope readers will find interesting, useful or at least provocative.

We first need to start thinking more systematically about disease natural history in light of the flood of new ideas and data coming into human biology and medicine. Our current thinking about biomedical science, models of health and disease and vocabulary all grew largely from advances in 19th century anatomy, histology and pathology, and mid-20th century biochemistry and physiology [65,66]. Several general precedents and new approaches provide conceptual and practical guidance on how to integrate new data and observations (see, e.g., [67,68,69]). These efforts start appropriately with James German’s suggestion, over 40 years ago, to focus on clonal evolution in vivo as a central biological process [2,70,71]. Thinking about somatic clonal evolution—the preferential expansion or contraction over time, in vivo, of mitotically related lineages of cells—may now be better enabled and explored by using ‘cellular reference trees’ [72]. These can be readily built from single cell-derived and related data sets, and have the potential to more directly reflect cellular, tissue and organismal states and trajectories than more conventional cell types and atlases (see, e.g., [73]). Trees also facilitate analyses of cell state over time, as modified by germline genetics, exposure and the interplay of genetic instability, somatic selection and senescence [74,75,76,77,78,79,80]. Current efforts to integrate cellular reference trees with universal cell embedding have the potential to provide a powerful and practically useful foundational model to advance A.I.-enabled RECQ biology and medicine [81] (see below). This combination should directly aid efforts to define the phenotypic ‘landscape’ or range of cell states and responses compatible with cell viability and function that is associated with any given genotype [82]. In part because the loss of WRN, BLM or other RECQ helicase protein functions confer often-strong cell-autonomous phenotypes, it is possible to use cell-based models to study many gene–gene, gene–drug and functional interactions at scale, and to identify ways to modify or compensate in part for loss of function [83]. The most interesting or promising results can then be further explored and modeled across different biological (spatial and temporal) scales [68,69] to understand the origins of cellular, tissue- and organismal-level phenotypes in the respective RECQ deficiency syndromes [47,48].

The slow temporal progression of the WS clinical phenotype is biologically interesting, with clear clinical as well as potential translational significance [4,84]. The mechanisms driving the development of the WS clinical phenotype may have very ‘shallow’ temporal slopes—small multipliers or rates of accumulation over time—and thus be difficult to identify against the backdrop of human genetic and phenotypic variation. One idea to make headway is to focus on cell types and tissues most strongly affected in WS, as these may possess the least functional reserve to compensate for loss of function. WS-specific and more general chronological age/disease-linked biomarkers are also needed to track progression and the response to interventions or therapies.

Identification of good biomarkers is a serious challenge facing all of biological and translational geroscience [85,86]. Routine lab values are a good place to start: abundant data already exist for many WS patients (e.g., for C-reactive protein and key inflammatory cytokines [87,88]), as these are often longitudinal data points that are linked to clinical management and outcomes. Better integration and analyses of these and related multi-omic data might give a palpable sense of how WS physiology and metabolism are ‘wired’ and respond to specific perturbations or treatments [89]. Deep metabolomic ‘fingerprints’ of WS and BS, once established, would be immediately useful for patient management while facilitating research efforts focused on rates of aging and age-associated disease mechanisms [86,90]. WS-specific precedents already exist in the form of metabolomics data from mouse models of WS [91] that can be compared with complementary Human Metabolome Project data [92].

Abundant new data have also recently become available on human methylation ‘clocks’ that may capture and integrate chronological and biological age data [93,94,95]. These data provide an opportunity to re-visit and extend early results suggesting WS-specific methylation differences [93], and can now be complemented by organ-specific protein trajectories that can be tracked via plasma proteomics. Despite some controversy about the nature and utility of methylation and other epigenetic biomarkers (see, e.g., [96,97]), it will be particularly interesting to see if WS patients are a disease-specific subset of ‘multi-organ agers’, or have more specific and potentially interesting differences in specific organs such as the brain [98].

Clonal hematopoiesis (CH) may represent an opportunity to track disease progression: although not well-documented in WS or BS, it can be potentiated by genetic instability, and can confer an elevated risk of cancer and other clinically important cardiovascular, metabolic and endocrine diseases that contribute to excess or premature morbidity and mortality in WS and BS [99,100,101,102]. CH is one example of somatic mosaicism that is more widespread than initially appreciated and may serve as a reservoir of somatic genetic variation, driving or predisposing people to a wide range of developmental and acquired late phenotypes and disease risks. We are just beginning to appreciate this aspect of ‘noisy human biology’, and the potential of more sophisticated quantitative measures to provide biological insight as well as useful new biomarkers. For example, we know red blood cell glycophorin-A variants can be easily quantified in WS and BS patients [27,103], and could be supplemented by methods being used or developed to track and define somatic mutational load and dynamics over the human lifespan, as part of the NIH ‘Somatic Mosaicism Across Human Tissues’ (SMaHT) initiative [104].

The histologic subtype specificity of cancer in heritable syndromes, including the human RECQ deficiency syndromes, remains one of the largest unsolved questions in cancer biology [105,106]. Combined analyses of syndrome-specific data that integrate germline and somatic genetic variation with cancer type-specific risk and alterations [76] are beginning to provide some insight into this question [107]. For example, recent work on defective telomere maintenance syndromes is leading us back to examine the role of telomere maintenance and metabolism in WS. Telomeres were among the first substrates identified for the WRN protein [108], and recent results with defective telomere maintenance syndromes indicate that excessive telomere gain or loss can both trigger neoplasia or senescence in affected tissues [109,110]. Disrupted telomere structure or function may act directly to promote genetic instability or senescence signaling, or indirectly with loss immune-mediated cancer suppression pathways following perturbed telomere-to-mitochondria signaling. This type of signaling could be mediated by ZBP1 [111], with leakage of mtDNA fragments into the cytoplasm triggering a potent pro-inflammatory IFN-1-dependent response [112,113]. It is of note that mitochondrial-nuclear signaling pathways also appear to be important determinants of longevity [114,115], as well as tumorigenesis [116,117] and disease risk. Mitochondrial control of cGAS-STING activity and signaling can be either tumor-promoting or inhibitory, depending on the context [118,119].

Other clues are suggested by previous unexplained results. For example, we identified a remarkably strong and statistically highly significant gene expression signature in WS patient-derived fibroblasts. This consisted of coordinate over-expression of the mitochondrial—but not cytoplasmic—tRNA synthetases [48], a chronic metabolic perturbation that could feed forward to trigger mitochondrial dysfunction, compromise mitochondrial metabolic activity and mtDNA maintenance [116,117,119] with initiation of disease-promoting cascades as outlined above. None of these connections has been critically explored as yet in WS, so there is ample scope for new discoveries of general significance that also provide new insight into WS disease pathogenesis.

**Figure 6:**
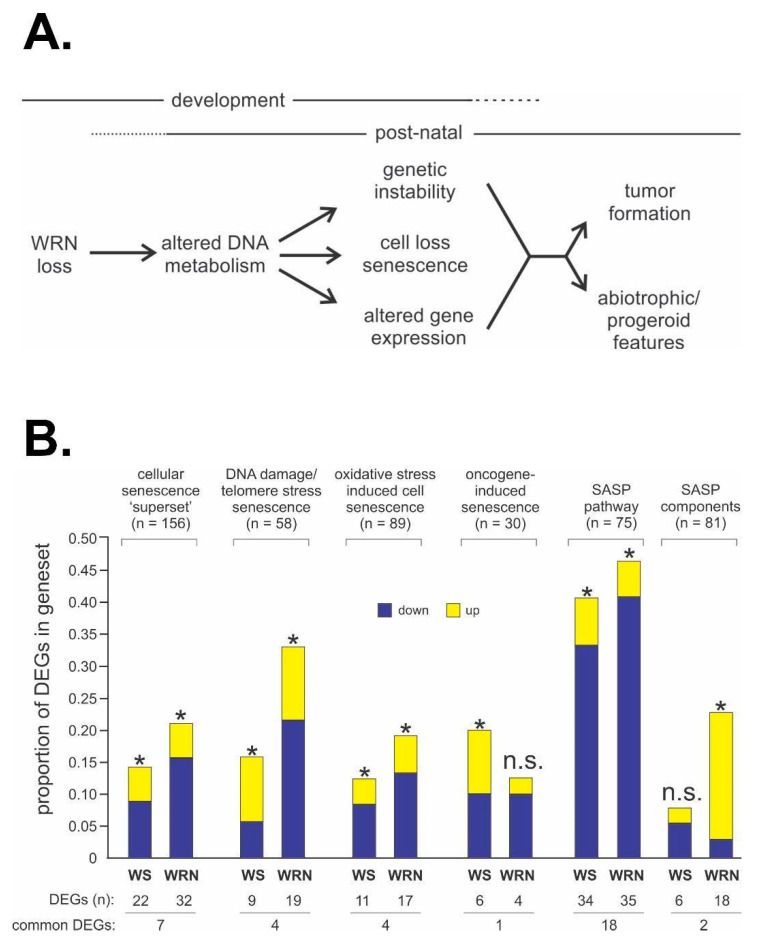
WS disease pathogenesis and potential mechanistic pathways and drivers. (**A**) Conceptual model emphasizing the development of tissue- and organism-level WS phenotypes as the outcome of disrupted cell structure and function driven by altered DNA metabolism after germline loss of WRN function. (**B**) Both acute WRN depletion and germline loss of WRN perturb expression of key genes and pathways associated with the response to DNA damage and with senescence, albeit in different ways. Differentially expressed genes (DEGs) in gene sets that were significantly altered in WRN-depleted (WS bars) and WS patient (WRN bars) cells versus all DEGs are indicated, with significant enrichment or depletion indicated by an asterisk (*). n.s. = not statistically significant. Source notes: Panel (**A**) is a global model originally and continually proposed and published in different forms by the author since the mid-1990s. Panel (**B**) is Figure 4 from Tang et al. [48].

Environmental drivers or modifiers of disease risk and penetrance have received comparatively little attention in WS, BS and most other heritable cancer predisposition syndromes (save for the clearly established role of UV light in the excision repair deficiency syndromes such as xeroderma pigmentosum). The RECQ deficiency syndromes and other cancer predisposition syndromes represent sensitized human organismal genotypes, in which environmental disease drivers should be more easily identified than in the general population [120,121]. The much more common heterozygous carrier state for pathogenic RECQ variants may have particular epidemiologic importance: it could, for example, heighten sensitivity to common environmental exposures, especially genotoxins [122], in ways not otherwise easily identified by looking only for canonical WS or BS clinical phenotypes. This work plays directly into our growing appreciation of the complexity of cancer, and how deciphering the complexity of cancer as a systemic disease process will provide both new biological insight and identify new and better ways to prevent and treat cancer [123]. The ability to integrate and explore these different types of data across different temporal and spatial scales will be powerfully amplified by AI and machine learning-enabled approaches [124,125,126]. Low-hanging fruit is already in sight with the identification of new associations among incompletely mapped or annotated data starting from simple text-based queries [127], where even simple gene expression profiling data (e.g., Figure 6) can provide useful and revealing points of departure.

## 8. Are Werner and Bloom Syndromes ‘Treatable’ Disease States?

Are WS and BS potentially treatable disease states? The slow clinical progression of WS may paradoxically be an advantage here if we can find safe, simple and effective ways to modify the biology that drives disease risk and pathogenesis. A worthy grand challenge here would be to develop a sophisticated understanding of what parts of human biology and metabolism we can profitably tinker with: even imprecisely identified or understood disease mechanisms, if targetable, might be useful to delay or prevent disease. Some potential approaches discussed in more detail elsewhere include NAD precursor supplementation [117,128]; pathway-focused therapeutics such as metformin [129]; the use of statins and the corresponding current enthusiasm to substantially broaden uses of the GLP-1 receptor agonists (see, e.g., [130]); the growing list of potential senolytics [131]); and the growing list of other plausible interventions (see, e.g., [132]). Other intriguing approaches are already on the horizon—for example, synthetic, biology-enabled microbiome engineering to target and correct tissue-level defects or imbalances ‘in trans’ (see, e.g., [133]). Structural defects previously thought of as fixed might also be reversible, as suggested by results from Hutchinson–Guilford mouse in vivo gene editing [134]. These and many other plausible and exciting directions are coming rapidly into view, though all will have challenging paths to clinical implementation. Nonetheless, these collectively promise revealing and practically useful futures and offer less risky places to start than alternatives such as wholesale in vivo cellular reprogramming.

## 9. The Essential Role of Rare Disease Communities in Advancing Knowledge

**Figure 7:**
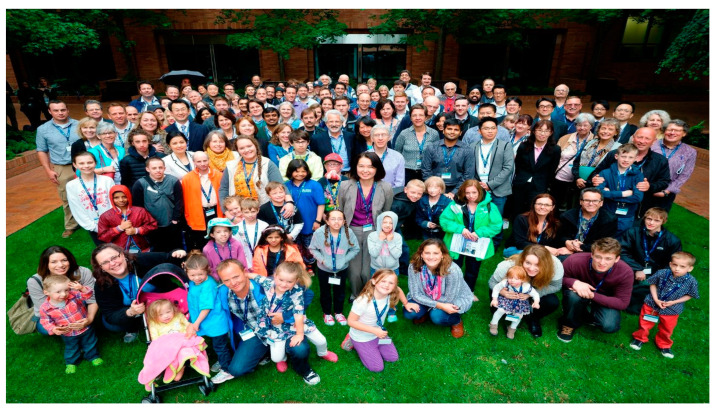
‘RECQ2016—Partnering for Progress’. This meeting brought over 120 patients, family members, and caregivers together with clinicians and scientists focused on BS, WS and Rothmund-Thomson syndrome at the Fred Hutchinson Cancer Center in Seattle for three days of presentations, discussion and socializing.

None of the scientific or clinical directions discussed above will gain any real traction unless pursued in conjunction with RECQ syndrome patients, caregivers and families. They are our essential partners in pursuing the opportunities outlined above, and we have the continuing obligation to them to return useful new knowledge as quickly as possible. It remains vitally important to bring clinicians, scientists, patients and families together to collectively identify and pursue shared research, clinical and translational goals. Two recent meetings to better establish this model were held in 2016 in Seattle (‘RECQ2016: Partnering for Progress’; see Figure 7), and in 2017 in Chiba, Japan ([135]).

An ongoing, related effort by all clinicians who care for RECQ deficiency syndrome patients must remain focused on continually improving supportive care. Atul Gawande has written perceptively about the value of this approach to chronic disease for individuals with cystic fibrosis and migraines [136,137]. We have accumulating evidence that this approach can also contribute to better health and longevity of WS and other RECQ deficiency syndrome patients [138,139,140]. These and related questions in RECQ syndrome clinical and translational medicine will continue to depend critically on dedicated, knowledgeable clinicians and clinician/scientists working in conjunction with patients, families and caregivers. My Seattle colleagues Junko Oshima, Fuki Hisama and the late George Martin contributed continuously to this effort by providing regularly updated information on WS as part of a publicly accessible GeneReviews record [4], and more recent, focused clinically-oriented guidance on WS patient care available via a WS record in ‘UptoDate’ and via corresponding efforts by Japanese colleagues (see, e.g., [140]).

## 10. Coda

I finish this perspective with two anecdotes that illustrate different sides of James German’s personality. The first occurred in 2009, at a RECQ Helicase meeting held in Chicago that included BS scientists, patients and family/caregiver communities. James German responded to a question at this meeting by stating ‘…we have no idea where the short stature of BS patients comes from’. This comment took me by surprise, in light of James’ extensive knowledge and familiarity with BS patients, the natural history of BS as a disease phenotype and new data related to BS cellular replication defects. My counter-argument to James was that even a cursory glance at BS patients provided a good—if provisional—answer to this question that could be experimentally verified: development as a process or program was normal (or largely normal) in individuals with BS, but was substrate-limited: too few good cells were generated during early embryogenesis to construct a normal-sized fetus, so the otherwise normal general program governing development simply scaled the output in light of available cells [141]. I remain surprised to this day that James was not more receptive to this line of reasoning, or ways to test this hypothesis using BS patient cells and tissue.

The second anecdote is decidedly more whimsical, and more telling of James’s sense of humor. After a 2001 meeting held in Tarrytown NY sponsored by the Ataxia-Telangiectasia Children’s Project, James offered to chauffeur several of us back into New York City in his station wagon. He considered the trip back a ‘teachable moment’ and thought we should be given some practical knowledge as part of the ride. In this instance the ‘practical knowledge’ was how to sneak onto Manhattan Island without paying any bridge or tunnel tolls. James took pride and satisfaction in demonstrating how to execute this quintessential ‘New York move’, to his amusement and that of his captive audience!

James German and others of his generation including George Martin, Arno Motulsky, Bob Miller and Haruo Sagano did so much to first stimulate interest in BS and WS as cancer predisposition syndromes that could reveal important aspects of human biology and disease. These early pioneers embodied what it means to be good ancestors: they directed our attention, gave us a sense of what might be possible, and showed us how to get started.
